# 4′-(1*H*-Imidazol-2-yl)-3′-[(1*H*-indol-3-yl)carbon­yl]-1′-methyl-2-oxo­spiro­[indoline-3,2′-pyrrolidine]-3′-carbo­nitrile 0.15-hydrate

**DOI:** 10.1107/S1600536813023246

**Published:** 2013-08-31

**Authors:** S. Antony Inglebert, Yuvaraj Arun, K. Sethusankar, Paramasivam T. Perumal

**Affiliations:** a Sri Ram Engineering College, Chennai 602 024, India; b Organic Chemistry Division, Central Leather Research Institute, Adyar, Chennai 600 020, India; c Department of Physics, RKM Vivekananda College (Autonomous), Chennai 600 004, India

## Abstract

In the title compound, C_25_H_20_N_6_O_2_·0.15H_2_O, the dihedral angles between the least-squares planes of the indole and pyrrolidine rings and between the oxindole and imidazole rings are 77.66 (7) and 45.31 (7)°, respectively. The pyrrolidine ring and the fused five-membered pyrrolidine ring of the oxindole moiety exhibit twisted conformations. The amide N atom is involved in both intra- and inter­molecular hydrogen bonding, having a bifurcated character. The mol­ecular structure is characterized by an intra­molecular N—H⋯O hydrogen bond, which generates an *S*(7) ring motif while an inter­molecular N—H⋯O hydrogen bond links the organic and solvent water mol­ecules. In the crystal, N—H⋯N hydrogen bonds generate a zigzag chain running parallel to *c-*axis direction. The H atoms of the solvent water mol­ecule were not located.

## Related literature

For background to indole derivatives and their biological activity, see: Rudrangi *et al.* (2011 ▶). For puckering parameters, see: Cremer & Pople (1975 ▶). For bond-length data, see: Allen *et al.* (1987 ▶). For graph-set notation, see: Bernstein *et al.* (1995 ▶). For a related structure, see: Inglebert *et al.* (2013 ▶). 
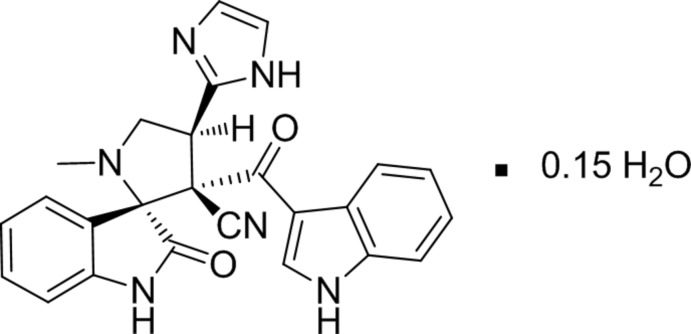


## Experimental

### 

#### Crystal data

C_25_H_20_N_6_O_2_·0.15H_2_O
*M* *_r_* = 439.17
Monoclinic, 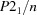

*a* = 8.650 (5) Å
*b* = 16.952 (5) Å
*c* = 14.438 (5) Å
β = 97.161 (5)°
*V* = 2100.6 (15) Å^3^
*Z* = 4
Mo *K*α radiation
μ = 0.09 mm^−1^
*T* = 295 K
0.35 × 0.30 × 0.25 mm

#### Data collection

Bruker Kappa APEXII CCD diffractometer
Absorption correction: multi-scan (*SADABS*; Bruker, 2008 ▶) *T*_min_ = 0.968, *T*_max_ = 0.977
22198 measured reflections
4819 independent reflections
3757 reflections with *I* > 2σ(*I*)
*R*_int_ = 0.024

#### Refinement

*R*[*F* ^2^ > 2σ(*F* ^2^)] = 0.040
*wR*(*F* ^2^) = 0.110
*S* = 1.03
4819 reflections
320 parameters
3 restraints
H atoms treated by a mixture of independent and constrained refinement
Δρ_max_ = 0.21 e Å^−3^
Δρ_min_ = −0.18 e Å^−3^

### 

Data collection: *APEX2* (Bruker, 2008 ▶); cell refinement: *SAINT* (Bruker, 2008 ▶); data reduction: *SAINT*; program(s) used to solve structure: *SHELXS97* (Sheldrick, 2008 ▶); program(s) used to refine structure: *SHELXL97* (Sheldrick, 2008 ▶); molecular graphics: *ORTEP-3 for Windows* (Farrugia, 2012 ▶); software used to prepare material for publication: *SHELXL97* and *PLATON* (Spek, 2009 ▶).

## Supplementary Material

Crystal structure: contains datablock(s) global, I. DOI: 10.1107/S1600536813023246/rk2411sup1.cif

Structure factors: contains datablock(s) I. DOI: 10.1107/S1600536813023246/rk2411Isup2.hkl

Additional supplementary materials:  crystallographic information; 3D view; checkCIF report

## Figures and Tables

**Table 1:** Hydrogen-bond geometry (Å, °)

*D*—H⋯*A*	*D*—H	H⋯*A*	*D*⋯*A*	*D*—H⋯*A*
N1—H1*A*⋯N5^i^	0.89 (1)	2.13 (1)	2.9889 (19)	164 (2)
N6—H6*A*⋯O1*W*	0.90 (1)	1.98 (2)	2.714 (8)	138 (2)
N6—H6*A*⋯O1	0.90 (1)	2.57 (2)	3.064 (2)	116 (2)

Symmetry code: (i) 
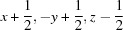
.

**Fig. 1.**
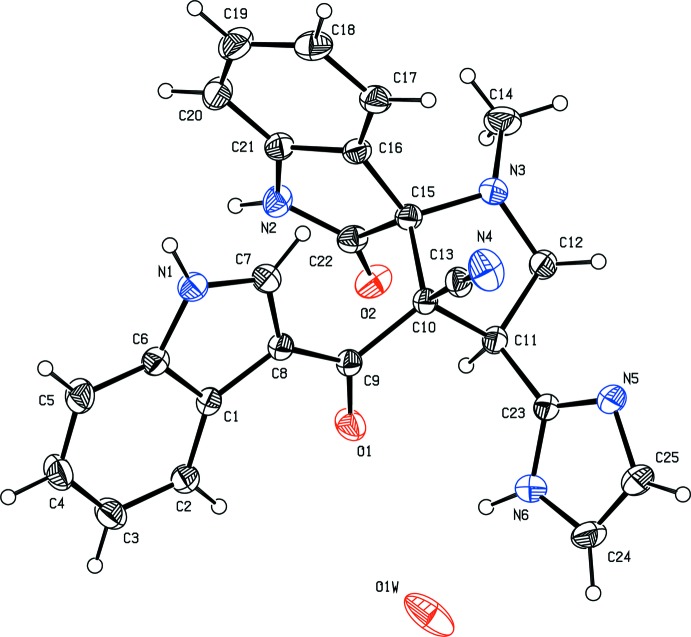
The asymmetric unit of the title compound with the atom numbering scheme. Displacement ellipsoids drawn at the 30% probability level. H atoms are presented as asmall spheres of arbitrary radius. H atoms of water molecule are not found.

**Fig. 2.**
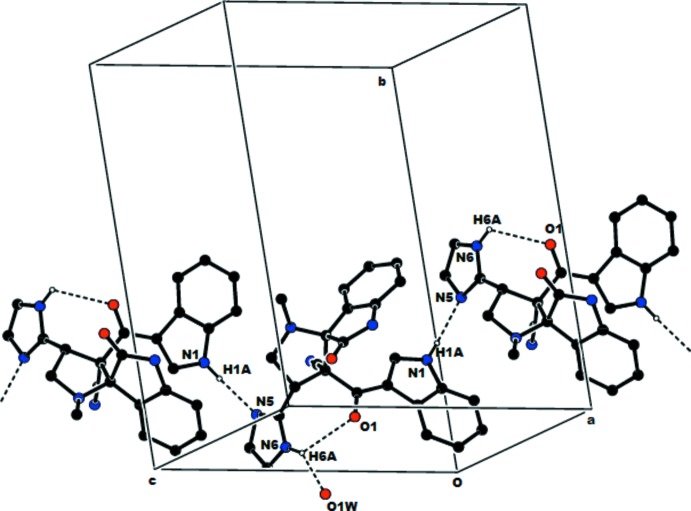
The packing diagram of the title compound viewed along the a axis. H atoms have omited for clarity.

## supplementary crystallographic information

### 1. Comment

Oxindoles are endogenous aromatic organic compounds that are found in the tissues and body fluids of mammals and in the natural products of some plants. Oxindoles exhibit an extensive range of biological effects, which include anticancer, anti–inflammatory, antiviral, antibacterial, antihypertensive and anticonvulsant activities. Oxindoles are also used to inhibit the replication of HIV and combat the infections that are caused by drug–resistant, drug–sensitive and mutant strains of HIV (Rudrangi *et al.*, 2011).

The title compound consists of a pyrrolidine ring connected to an imidazole at C11, an oxindole ring system at C15, a methyl group at N3 and an indole unit at C10 *via* carbonyl group. In addition, the asymmetric unit contains a 0.15 occupancy water molecule. The H atoms of the partial occupancy water molecules are neither located nor included in the refinement. The title structure exhibits structural similarities with the previously reported structure (Inglebert *et al.*, 2013).

The pyrrolidine ring adopts a twisted conformation with puckering parameters q(2) = 0.4351 (14)Å and φ(2) = 309.66 (19)°. Pyrrole ring in the oxindole unit also having twisted conformation with puckering parameters q(2) = 0.0931 (15)Å and φ(2) = 304.2 (9)°. The least square plane of oxindole unit makes dihedral angles of 78.50 (4)° and 44.28 (5)° with the pyrrolidine and imidazole, respectively. The indole unit is essentially planar - maximum deviation = 0.0138 (17)Å for the C2 atom] and is oriented at a least square plane that makes dihedral angles of 51.42 (4)°, 39.92 (4)° and 70.15 (4)°, with the oxindole unit, pyrrolidine and imidazole rings, respectively.

The cyano bond distance C13≡N2 agrees well with the reported value of 1.138 (7)Å (Allen *et al.*, 1987). The sum of the angle around atom N3 (340.14 (35)°) is in accordance with *sp^3^* hybridization. The amide N atom shows bifurcated intramolecular hydrogen bond (N—H···O) with an O atom of the carbonyl group and an intermolecuar hydrogen (N—H···O) bond with the 0.15 occupancy solvent water molecule. In addition, the classical intermolecular N—H···N hydrogen bonds generate a zigzag chain running parallel to *c* axis.

### 2. Experimental

A mixture of isatin (1 mmol), sarcosine (1.2 mmol) and 3-(1*H*-imidazol-2-yl)-2-(1*H*-indole-3-carbonyl)acrylonitrile (1 mmol) were refluxed in ethanol (30 ml). After completion of the reaction as evidenced by *TLC* analysis, the reaction mixture was poured into ice–water, the resulting solid was filtered off and purified by column chromatography using ethyl acetate : petroleum ether (6 : 4) as an eluent to afford pure product.

### 3. Refinement

Positions of hydrogen atoms were localized from the difference electron density maps and their distances were geometrically constrained. The H atoms bound to the C atoms were treated as riding atoms with d(C—H) = 0.93Å for aromatic H, d(C—H) = 0.97Å for methylene H with *U*_iso_(H) = 1.2*U*_eq_(C) and and d(C—H) = 0.96Å and *U*_iso_(H) = 1.5*U*_eq_(C) for methyl H atoms. The rotation angles for methyl groups were optimized by least squares. The N bonded H atoms were refined freely.

### Crystal data

**Table:**

C_25_H_20_N_6_O_2_·0.15H_2_O	*F*(000) = 916.8
*M**_r_* = 439.17	*D*_x_ = 1.389 Mg m^−^^3^
Monoclinic, *P*2_1_/*n*	Mo *K*α radiation, λ = 0.71073 Å
Hall symbol: -P 2yn	Cell parameters from 4819 reflections
*a* = 8.650 (5) Å	θ = 2.4–27.5°
*b* = 16.952 (5) Å	µ = 0.09 mm^−^^1^
*c* = 14.438 (5) Å	*T* = 295 K
β = 97.161 (5)°	Block, colourless
*V* = 2100.6 (15) Å^3^	0.35 × 0.30 × 0.25 mm
*Z* = 4	

### Data collection

**Table:**

Bruker Kappa APEXII CCD diffractometer	4819 independent reflections
Radiation source: fine–focus sealed tube	3757 reflections with *I* > 2σ(*I*)
Graphite monochromator	*R*_int_ = 0.024
ω and φ scans	θ_max_ = 27.5°, θ_min_ = 2.4°
Absorption correction: multi-scan (*SADABS*; Bruker, 2008)	*h* = −11→10
*T*_min_ = 0.968, *T*_max_ = 0.977	*k* = −21→22
22198 measured reflections	*l* = −18→18

### Refinement

**Table:**

Refinement on *F*^2^	Primary atom site location: structure-invariant direct methods
Least-squares matrix: full	Secondary atom site location: difference Fourier map
*R*[*F*^2^ > 2σ(*F*^2^)] = 0.040	Hydrogen site location: inferred from neighbouring sites
*wR*(*F*^2^) = 0.110	H atoms treated by a mixture of independent and constrained refinement
*S* = 1.03	*w* = 1/[σ^2^(*F*_o_^2^) + (0.0524*P*)^2^ + 0.5356*P*] where *P* = (*F*_o_^2^ + 2*F*_c_^2^)/3
4819 reflections	(Δ/σ)_max_ < 0.001
320 parameters	Δρ_max_ = 0.21 e Å^−^^3^
3 restraints	Δρ_min_ = −0.18 e Å^−^^3^

### Special details

**Table:**

Geometry. All s.u.'s (except the s.u. in the dihedral angle between two l.s. planes) are estimated using the full covariance matrix. The cell s.u.'s are taken into account individually in the estimation of s.u.'s in distances, angles and torsion angles; correlations between s.u.'s in cell parameters are only used when they are defined by crystal symmetry. An approximate (isotropic) treatment of cell s.u.'s is used for estimating s.u.'s involving l.s. planes.
Refinement. Refinement of *F*^2^ against ALL reflections. The weighted *R*–factor *wR* and goodness of fit *S* are based on *F*^2^, conventional *R*–factors *R* are based on *F*, with *F* set to zero for negative *F*^2^. The threshold expression of *F*^2^ > σ(*F*^2^) is used only for calculating *R*–factors(gt) *etc*. and is not relevant to the choice of reflections for refinement. *R*–factors based on *F*^2^ are statistically about twice as large as those based on *F*, and *R*–factors based on ALL data will be even larger.

### Fractional atomic coordinates and isotropic or equivalent isotropic displacement parameters (Å^2^)

**Table:**

	*x*	*y*	*z*	*U*_iso_*/*U*_eq_	Occ. (<1)
O1	0.37937 (14)	0.07447 (6)	0.49050 (7)	0.0487 (3)	
O2	0.60471 (12)	0.18182 (6)	0.64371 (8)	0.0489 (3)	
N3	0.33938 (14)	0.30031 (7)	0.63558 (8)	0.0366 (3)	
N2	0.66582 (14)	0.25656 (8)	0.52028 (9)	0.0406 (3)	
N1	0.38442 (14)	0.21594 (7)	0.22422 (8)	0.0380 (3)	
C15	0.40978 (15)	0.26985 (7)	0.55702 (9)	0.0315 (3)	
C10	0.28828 (14)	0.20355 (7)	0.52052 (9)	0.0292 (3)	
N6	0.12101 (15)	0.03867 (7)	0.61150 (9)	0.0432 (3)	
N5	−0.02773 (15)	0.14222 (8)	0.61946 (11)	0.0508 (3)	
C1	0.42119 (15)	0.09829 (8)	0.29638 (9)	0.0318 (3)	
C16	0.44630 (15)	0.33152 (8)	0.48826 (9)	0.0340 (3)	
C11	0.25875 (15)	0.16641 (8)	0.61571 (9)	0.0326 (3)	
H11	0.3466	0.1309	0.6343	0.039*	
C6	0.43006 (16)	0.13884 (8)	0.21219 (9)	0.0352 (3)	
C9	0.34886 (14)	0.13897 (8)	0.45599 (9)	0.0313 (3)	
C8	0.36716 (15)	0.15545 (8)	0.36003 (9)	0.0316 (3)	
C12	0.27540 (17)	0.23523 (8)	0.68508 (10)	0.0399 (3)	
H12A	0.1749	0.2494	0.7034	0.048*	
H12B	0.3452	0.2213	0.7406	0.048*	
C7	0.34692 (16)	0.22545 (8)	0.31089 (9)	0.0359 (3)	
H7	0.3123	0.2724	0.3345	0.043*	
C21	0.59667 (16)	0.31921 (8)	0.46655 (10)	0.0368 (3)	
C2	0.46065 (17)	0.01848 (8)	0.30176 (10)	0.0386 (3)	
H2	0.4569	−0.0096	0.3568	0.046*	
C22	0.57128 (15)	0.22878 (8)	0.58113 (10)	0.0359 (3)	
C13	0.14541 (16)	0.24026 (8)	0.47441 (9)	0.0361 (3)	
C3	0.50525 (19)	−0.01783 (9)	0.22397 (11)	0.0478 (4)	
H3	0.5299	−0.0712	0.2266	0.057*	
C23	0.11522 (16)	0.11799 (8)	0.61483 (9)	0.0351 (3)	
C19	0.5689 (2)	0.42550 (10)	0.36056 (12)	0.0552 (4)	
H19	0.6079	0.4569	0.3160	0.066*	
N4	0.03167 (16)	0.26634 (9)	0.43965 (10)	0.0581 (4)	
C5	0.47677 (19)	0.10239 (10)	0.13400 (10)	0.0467 (4)	
H5	0.4824	0.1302	0.0790	0.056*	
C20	0.66026 (19)	0.36546 (10)	0.40259 (11)	0.0476 (4)	
H20	0.7607	0.3566	0.3883	0.057*	
C4	0.5143 (2)	0.02372 (10)	0.14131 (11)	0.0512 (4)	
H4	0.5462	−0.0023	0.0903	0.061*	
C17	0.35815 (18)	0.39336 (8)	0.44843 (11)	0.0424 (3)	
H17	0.2594	0.4036	0.4648	0.051*	
C18	0.4207 (2)	0.44015 (9)	0.38310 (12)	0.0524 (4)	
H18	0.3625	0.4815	0.3544	0.063*	
C25	−0.1158 (2)	0.07419 (10)	0.61833 (14)	0.0584 (5)	
H25	−0.2227	0.0728	0.6207	0.070*	
C14	0.4324 (2)	0.35557 (10)	0.69609 (12)	0.0555 (4)	
H14A	0.5180	0.3281	0.7306	0.083*	
H14B	0.3690	0.3790	0.7387	0.083*	
H14C	0.4718	0.3961	0.6589	0.083*	
C24	−0.0261 (2)	0.01074 (10)	0.61340 (12)	0.0513 (4)	
H24	−0.0576	−0.0418	0.6116	0.062*	
O1W	0.2610 (10)	−0.0987 (6)	0.5729 (7)	0.082 (3)	0.15
H2A	0.7632 (12)	0.2407 (9)	0.5205 (12)	0.052 (5)*	
H1A	0.393 (2)	0.2564 (8)	0.1861 (11)	0.061 (5)*	
H6A	0.2060 (17)	0.0112 (11)	0.6021 (15)	0.075 (6)*	

### Atomic displacement parameters (Å^2^)

**Table:**

	*U*^11^	*U*^22^	*U*^33^	*U*^12^	*U*^13^	*U*^23^
O1	0.0721 (8)	0.0369 (6)	0.0403 (5)	0.0139 (5)	0.0190 (5)	0.0060 (4)
O2	0.0363 (6)	0.0479 (6)	0.0605 (7)	0.0013 (5)	−0.0018 (5)	0.0177 (5)
N3	0.0386 (6)	0.0330 (6)	0.0384 (6)	−0.0033 (5)	0.0063 (5)	−0.0054 (5)
N2	0.0267 (6)	0.0464 (7)	0.0486 (7)	0.0010 (5)	0.0047 (5)	0.0038 (5)
N1	0.0448 (7)	0.0351 (6)	0.0344 (6)	0.0017 (5)	0.0053 (5)	0.0064 (5)
C15	0.0275 (6)	0.0294 (6)	0.0374 (6)	−0.0001 (5)	0.0035 (5)	0.0001 (5)
C10	0.0257 (6)	0.0300 (6)	0.0324 (6)	0.0002 (5)	0.0053 (5)	0.0000 (5)
N6	0.0462 (8)	0.0346 (7)	0.0494 (7)	−0.0044 (6)	0.0088 (6)	−0.0035 (5)
N5	0.0386 (7)	0.0406 (7)	0.0776 (9)	−0.0088 (6)	0.0247 (6)	−0.0085 (6)
C1	0.0292 (6)	0.0354 (7)	0.0315 (6)	−0.0020 (5)	0.0064 (5)	0.0007 (5)
C16	0.0333 (7)	0.0297 (7)	0.0389 (7)	−0.0044 (5)	0.0041 (5)	−0.0004 (5)
C11	0.0324 (7)	0.0348 (7)	0.0316 (6)	−0.0025 (5)	0.0077 (5)	−0.0014 (5)
C6	0.0350 (7)	0.0360 (7)	0.0350 (7)	−0.0007 (6)	0.0059 (5)	0.0028 (5)
C9	0.0294 (6)	0.0312 (7)	0.0339 (6)	0.0005 (5)	0.0060 (5)	0.0006 (5)
C8	0.0295 (6)	0.0323 (7)	0.0334 (6)	−0.0004 (5)	0.0059 (5)	0.0003 (5)
C12	0.0425 (8)	0.0419 (8)	0.0364 (7)	−0.0086 (6)	0.0098 (6)	−0.0073 (6)
C7	0.0355 (7)	0.0358 (7)	0.0361 (7)	0.0022 (6)	0.0027 (5)	−0.0003 (5)
C21	0.0346 (7)	0.0364 (7)	0.0393 (7)	−0.0054 (6)	0.0043 (5)	−0.0027 (6)
C2	0.0435 (8)	0.0353 (7)	0.0384 (7)	0.0014 (6)	0.0103 (6)	0.0040 (6)
C22	0.0288 (7)	0.0349 (7)	0.0428 (7)	−0.0029 (5)	−0.0006 (5)	0.0002 (6)
C13	0.0313 (7)	0.0401 (8)	0.0371 (7)	0.0006 (6)	0.0054 (5)	−0.0046 (6)
C3	0.0597 (10)	0.0378 (8)	0.0474 (8)	0.0095 (7)	0.0127 (7)	−0.0012 (6)
C23	0.0393 (8)	0.0335 (7)	0.0343 (6)	−0.0058 (6)	0.0117 (5)	−0.0038 (5)
C19	0.0660 (11)	0.0504 (10)	0.0516 (9)	−0.0112 (8)	0.0166 (8)	0.0112 (7)
N4	0.0386 (7)	0.0737 (10)	0.0595 (8)	0.0149 (7)	−0.0037 (6)	−0.0056 (7)
C5	0.0568 (9)	0.0520 (9)	0.0331 (7)	0.0037 (7)	0.0129 (6)	0.0051 (6)
C20	0.0454 (9)	0.0515 (9)	0.0480 (8)	−0.0071 (7)	0.0141 (7)	0.0021 (7)
C4	0.0645 (11)	0.0526 (10)	0.0391 (8)	0.0101 (8)	0.0165 (7)	−0.0054 (7)
C17	0.0436 (8)	0.0324 (7)	0.0513 (8)	0.0022 (6)	0.0066 (6)	0.0023 (6)
C18	0.0642 (11)	0.0359 (8)	0.0564 (9)	0.0003 (7)	0.0048 (8)	0.0115 (7)
C25	0.0441 (9)	0.0502 (10)	0.0849 (13)	−0.0177 (8)	0.0239 (8)	−0.0101 (9)
C14	0.0714 (11)	0.0435 (9)	0.0515 (9)	−0.0174 (8)	0.0078 (8)	−0.0134 (7)
C24	0.0586 (10)	0.0394 (8)	0.0569 (9)	−0.0187 (8)	0.0116 (7)	−0.0048 (7)
O1W	0.063 (5)	0.070 (6)	0.102 (7)	0.036 (5)	−0.028 (5)	−0.039 (5)

### Geometric parameters (Å, º)

**Table:**

O1—C9	1.2168 (16)	C11—H11	0.9800
O2—C22	1.2119 (17)	C6—C5	1.390 (2)
N3—C15	1.4478 (17)	C9—C8	1.4413 (18)
N3—C14	1.4538 (19)	C8—C7	1.3826 (19)
N3—C12	1.4604 (18)	C12—H12A	0.9700
N2—C22	1.3570 (19)	C12—H12B	0.9700
N2—C21	1.4043 (19)	C7—H7	0.9300
N2—H2A	0.884 (9)	C21—C20	1.377 (2)
N1—C7	1.3410 (18)	C2—C3	1.377 (2)
N1—C6	1.3825 (19)	C2—H2	0.9300
N1—H1A	0.888 (9)	C13—N4	1.1367 (19)
C15—C16	1.5022 (18)	C3—C4	1.396 (2)
C15—C22	1.561 (2)	C3—H3	0.9300
C15—C10	1.5835 (18)	C19—C20	1.382 (2)
C10—C13	1.4671 (19)	C19—C18	1.383 (3)
C10—C11	1.5615 (18)	C19—H19	0.9300
C10—C9	1.5693 (18)	C5—C4	1.373 (2)
N6—C23	1.3467 (19)	C5—H5	0.9300
N6—C24	1.361 (2)	C20—H20	0.9300
N6—H6A	0.895 (9)	C4—H4	0.9300
N5—C23	1.313 (2)	C17—C18	1.393 (2)
N5—C25	1.381 (2)	C17—H17	0.9300
C1—C2	1.395 (2)	C18—H18	0.9300
C1—C6	1.4068 (18)	C25—C24	1.333 (2)
C1—C8	1.4522 (18)	C25—H25	0.9300
C16—C17	1.3790 (19)	C14—H14A	0.9600
C16—C21	1.391 (2)	C14—H14B	0.9600
C11—C23	1.4871 (19)	C14—H14C	0.9600
C11—C12	1.5325 (19)	C24—H24	0.9300
			
C15—N3—C14	116.43 (12)	H12A—C12—H12B	108.8
C15—N3—C12	109.63 (11)	N1—C7—C8	110.23 (12)
C14—N3—C12	114.08 (12)	N1—C7—H7	124.9
C22—N2—C21	111.75 (12)	C8—C7—H7	124.9
C22—N2—H2A	122.9 (11)	C20—C21—C16	121.90 (14)
C21—N2—H2A	124.9 (11)	C20—C21—N2	128.52 (14)
C7—N1—C6	109.69 (11)	C16—C21—N2	109.56 (12)
C7—N1—H1A	122.3 (12)	C3—C2—C1	118.65 (13)
C6—N1—H1A	127.3 (12)	C3—C2—H2	120.7
N3—C15—C16	114.40 (11)	C1—C2—H2	120.7
N3—C15—C22	115.88 (11)	O2—C22—N2	127.13 (13)
C16—C15—C22	101.64 (10)	O2—C22—C15	125.67 (13)
N3—C15—C10	100.80 (10)	N2—C22—C15	107.20 (12)
C16—C15—C10	117.35 (11)	N4—C13—C10	177.44 (16)
C22—C15—C10	107.23 (10)	C2—C3—C4	121.51 (14)
C13—C10—C11	110.45 (11)	C2—C3—H3	119.2
C13—C10—C9	110.56 (10)	C4—C3—H3	119.2
C11—C10—C9	110.16 (10)	N5—C23—N6	110.71 (12)
C13—C10—C15	109.67 (11)	N5—C23—C11	128.15 (13)
C11—C10—C15	99.66 (10)	N6—C23—C11	121.11 (13)
C9—C10—C15	115.84 (10)	C20—C19—C18	121.66 (15)
C23—N6—C24	107.91 (13)	C20—C19—H19	119.2
C23—N6—H6A	124.1 (14)	C18—C19—H19	119.2
C24—N6—H6A	127.6 (14)	C4—C5—C6	117.31 (13)
C23—N5—C25	105.06 (14)	C4—C5—H5	121.3
C2—C1—C6	118.86 (12)	C6—C5—H5	121.3
C2—C1—C8	135.04 (12)	C21—C20—C19	117.34 (15)
C6—C1—C8	106.10 (12)	C21—C20—H20	121.3
C17—C16—C21	120.26 (13)	C19—C20—H20	121.3
C17—C16—C15	130.83 (13)	C5—C4—C3	121.18 (14)
C21—C16—C15	108.91 (12)	C5—C4—H4	119.4
C23—C11—C12	115.86 (11)	C3—C4—H4	119.4
C23—C11—C10	116.30 (11)	C16—C17—C18	118.30 (15)
C12—C11—C10	104.83 (11)	C16—C17—H17	120.9
C23—C11—H11	106.4	C18—C17—H17	120.9
C12—C11—H11	106.4	C19—C18—C17	120.45 (15)
C10—C11—H11	106.4	C19—C18—H18	119.8
N1—C6—C5	129.68 (13)	C17—C18—H18	119.8
N1—C6—C1	107.83 (12)	C24—C25—N5	110.53 (15)
C5—C6—C1	122.49 (13)	C24—C25—H25	124.7
O1—C9—C8	121.56 (12)	N5—C25—H25	124.7
O1—C9—C10	117.08 (11)	N3—C14—H14A	109.5
C8—C9—C10	121.35 (11)	N3—C14—H14B	109.5
C7—C8—C9	129.61 (12)	H14A—C14—H14B	109.5
C7—C8—C1	106.14 (11)	N3—C14—H14C	109.5
C9—C8—C1	124.17 (12)	H14A—C14—H14C	109.5
N3—C12—C11	105.41 (11)	H14B—C14—H14C	109.5
N3—C12—H12A	110.7	C25—C24—N6	105.79 (14)
C11—C12—H12A	110.7	C25—C24—H24	127.1
N3—C12—H12B	110.7	N6—C24—H24	127.1
C11—C12—H12B	110.7		
			
C14—N3—C15—C16	−62.11 (16)	C15—N3—C12—C11	−19.05 (14)
C12—N3—C15—C16	166.52 (11)	C14—N3—C12—C11	−151.66 (13)
C14—N3—C15—C22	55.69 (16)	C23—C11—C12—N3	−139.92 (12)
C12—N3—C15—C22	−75.69 (14)	C10—C11—C12—N3	−10.31 (14)
C14—N3—C15—C10	171.01 (12)	C6—N1—C7—C8	0.44 (16)
C12—N3—C15—C10	39.64 (13)	C9—C8—C7—N1	176.60 (13)
N3—C15—C10—C13	72.92 (12)	C1—C8—C7—N1	−0.36 (15)
C16—C15—C10—C13	−51.98 (14)	C17—C16—C21—C20	−2.7 (2)
C22—C15—C10—C13	−165.45 (11)	C15—C16—C21—C20	177.84 (13)
N3—C15—C10—C11	−43.01 (11)	C17—C16—C21—N2	175.99 (12)
C16—C15—C10—C11	−167.91 (11)	C15—C16—C21—N2	−3.47 (15)
C22—C15—C10—C11	78.62 (12)	C22—N2—C21—C20	175.24 (14)
N3—C15—C10—C9	−161.10 (10)	C22—N2—C21—C16	−3.34 (16)
C16—C15—C10—C9	74.00 (14)	C6—C1—C2—C3	−0.7 (2)
C22—C15—C10—C9	−39.47 (14)	C8—C1—C2—C3	178.51 (15)
N3—C15—C16—C17	−45.93 (19)	C21—N2—C22—O2	−170.64 (14)
C22—C15—C16—C17	−171.58 (14)	C21—N2—C22—C15	8.35 (15)
C10—C15—C16—C17	71.86 (19)	N3—C15—C22—O2	44.68 (19)
N3—C15—C16—C21	133.45 (12)	C16—C15—C22—O2	169.35 (14)
C22—C15—C16—C21	7.80 (14)	C10—C15—C22—O2	−66.94 (17)
C10—C15—C16—C21	−108.75 (13)	N3—C15—C22—N2	−134.34 (12)
C13—C10—C11—C23	46.36 (16)	C16—C15—C22—N2	−9.67 (13)
C9—C10—C11—C23	−76.07 (14)	C10—C15—C22—N2	114.04 (12)
C15—C10—C11—C23	161.69 (11)	C1—C2—C3—C4	1.2 (2)
C13—C10—C11—C12	−82.99 (13)	C25—N5—C23—N6	−0.39 (18)
C9—C10—C11—C12	154.58 (11)	C25—N5—C23—C11	−178.58 (14)
C15—C10—C11—C12	32.35 (12)	C24—N6—C23—N5	0.46 (17)
C7—N1—C6—C5	179.16 (15)	C24—N6—C23—C11	178.79 (12)
C7—N1—C6—C1	−0.33 (16)	C12—C11—C23—N5	41.7 (2)
C2—C1—C6—N1	179.51 (12)	C10—C11—C23—N5	−82.13 (18)
C8—C1—C6—N1	0.10 (15)	C12—C11—C23—N6	−136.32 (14)
C2—C1—C6—C5	0.0 (2)	C10—C11—C23—N6	99.85 (15)
C8—C1—C6—C5	−179.43 (13)	N1—C6—C5—C4	−179.14 (15)
C13—C10—C9—O1	−129.88 (13)	C1—C6—C5—C4	0.3 (2)
C11—C10—C9—O1	−7.51 (16)	C16—C21—C20—C19	0.3 (2)
C15—C10—C9—O1	104.60 (14)	N2—C21—C20—C19	−178.08 (15)
C13—C10—C9—C8	50.01 (16)	C18—C19—C20—C21	1.5 (3)
C11—C10—C9—C8	172.37 (11)	C6—C5—C4—C3	0.2 (3)
C15—C10—C9—C8	−75.52 (15)	C2—C3—C4—C5	−0.9 (3)
O1—C9—C8—C7	−176.01 (14)	C21—C16—C17—C18	3.1 (2)
C10—C9—C8—C7	4.1 (2)	C15—C16—C17—C18	−177.58 (14)
O1—C9—C8—C1	0.5 (2)	C20—C19—C18—C17	−1.1 (3)
C10—C9—C8—C1	−179.41 (11)	C16—C17—C18—C19	−1.3 (2)
C2—C1—C8—C7	−179.12 (15)	C23—N5—C25—C24	0.2 (2)
C6—C1—C8—C7	0.15 (14)	N5—C25—C24—N6	0.1 (2)
C2—C1—C8—C9	3.7 (2)	C23—N6—C24—C25	−0.32 (19)
C6—C1—C8—C9	−177.02 (12)		

### Hydrogen-bond geometry (Å, º)

**Table:**

*D*—H···*A*	*D*—H	H···*A*	*D*···*A*	*D*—H···*A*
N1—H1*A*···N5^i^	0.89 (1)	2.13 (1)	2.9889 (19)	164 (2)
N6—H6*A*···O1*W*	0.90 (1)	1.98 (2)	2.714 (8)	138 (2)
N6—H6*A*···O1	0.90 (1)	2.57 (2)	3.064 (2)	116 (2)

Symmetry code: (i) *x*+1/2, −*y*+1/2, *z*−1/2.
